# High Density Arrayed Ni/NiO Core-shell Nanospheres Evenly Distributed on Graphene for Ultrahigh Performance Supercapacitor

**DOI:** 10.1038/s41598-017-17899-6

**Published:** 2017-12-18

**Authors:** Fanggang Liu, Xiaobing Wang, Jin Hao, Shuang Han, Jianshe Lian, Qing Jiang

**Affiliations:** Key Laboratory of Automobile Materials, Ministry of Education, and Department of Materials Science and Engineering, Jilin University, Changchun, 130022 P.R. China

## Abstract

A novel NiO/Ni/RGO three-dimensional core-shell architecture consisting of Ni nanoparticles as core, NiO as shell and reduced graphene oxide (RGO) as conductivity layer, has been constructed by redox reactions with hydrothermal method and heat treatment. High density arrayed nickel nanoparticles (20 nm diameter) semi-coated by a 3 nm thick layer of NiO are evenly distributed on the surface of graphene. This elaborate design not only uses abundant NiO surfaces to provide a wealth of active sites, but also bridges electrochemical active NiO shell and graphene by Ni core to construct an interconnected 3D conductive network. Since both electrochemical activity and excellent conductivity are reserved in this Ni/NiO core-shell/graphene layer 3D structure, the as-prepared electrode material exhibits an extremely high specific capacitance (2048.3 F g^−1^ at current density of 1 A g^−1^) and excellent cycle stability (77.8% capacitance retention after 10000 cycles at current density of 50 A g^−1^). The novel method presented here is easy and effective and would provide reference for the preparation of other high performance supercapacitor electrodes.

## Introduction

With the excessive consumption of traditional energy resources as well as fast-growing market of portable electronics and hybrid electric vehicles, development of high efficiency and sustainable energy storage systems, such as lithium ion batteries, electrochemical capacitors, has been put forward^1–4^. Supercapacitors, owing to its ultrahigh power density, fast charge-discharge property and long cycle life, arouse great interest^5,6^. Generally, supercapacitors can be classified to electric double layer capacitors (EDLCs) and pseudocapacitors based on different charge storage mechanism. EDLCs exhibit high power density and long cycle life, but low specific capacitance. In contrast, pseudocapacitors show higher specific capacitance and better energy density, but the low utilization rate of active materials and poor conductivity limited its application^7–9^. In recent years, transition metal oxides by virtue of high theoretical specific capacitance and electrochemical stability have been studied as electrode materials, such as NiO, MoO_3_, MnO_2_, RuO_2_^10–13^. Therein, NiO possess high theoretical capacitance (2584 F g^−1^), low cost and excellent chemical/thermal stability and are considered to be a promising electrode material^14,15^. Many efforts have been done to prepare NiO based supercapacitors^16,17^, but there is still a certain gap with its theoretical capacitance. Initially, the diffusion distance of electrolytes into NiO electrodes is limited, thus for the bulk structure lacking the diffusion channel, only the surface portion participates in the electrochemical reaction, leading to a less than optimal performance. Furthermore, the poor electrical conductivity of NiO would restrict the fast exchange of electrons. Additionally, any attempt to increase the mass loading usually results in an increase of material thickness, which consequently leads to more active materials being buried and increases the length of active materials away from conductivity base, and correspondingly lower overall efficiencies.

Hence, the thinner thickness of NiO layer, the higher utilization rate of NiO active material. So the NiO based core-shell structures have been widely prepared for supercapacitors, in order to efficiently utilize their rich surface active sites. But the present two kinds of core-shell structure still have some defects. For the core-shell structures fully covered with NiO active material, although abundant sufficient chemically active sites are presented, the electron exchange between every two neighboring core-shell structures was still blocked by at least two layer low conductivity NiO shell^18^. For the core-shell structures coated a layer of conductive material (such as carbon material) outside the NiO active material, the conductivity does increase significantly, the active material is isolated from the electrolyte. For example, the shell-core structure formed by amorphous carbon coated mesoporous NiO through a hydrothermal method exhibited a specific capacitance of 931 F g^−1^ at 2 A g^−1^ ^19^. So, for an expected NiO based core-shell structure as electrode material, increasing the electrochemical active surface while enhancing the conductivity are the key points to improve its electrochemical performance. But so far, these two necessary advantages haven’t been well coordinated.

In the present work, we consider a semi-coated core-shell structure for NiO based materials, the core metal is partially coated by active NiO shell and partially connected to the conductive matrix material, to realize the both the electrochemical activity and electron transfer.

As excellent electric conductive matrix material, Pt has been used as the electron collector to connected the NiO core-shell structure, which exhibits both high energy and power densities^20^. However, Pt is more expensive and may presents weak contact to the NiO^21^. Therefore, a cheap and efficient conductive material that can bring atomic bonding with both NiO shell and metal core is a prioritized proposal. Recent years, graphene attracted much attention owing to its outstanding electric conductivity, high specific surface area and good chemical stability^22–24^. Various approaches to hybrid graphene with nickel oxide proved that it’s helpful to improve the conductivity of supercapacitors^25–27^. However, due to the limitation of the amount and distribution of functional groups on graphene, the problem of uneven and low density distribution of NiO particles on graphene arise in NiO/graphene composites, which makes the NiO/graphene electrode materials lacking of sufficient reactive sites and continuous ion diffusion channels. Therefore, an exquisite design of 3D structure of NiO/metal/ graphene to realize fast transfer among high capacitance NiO unites and improve conductivity is critical for ideal NiO based supercapacitor materials.

Herein, we designed a novel NiO/Ni/RGO three-dimensional semi-coated core-shell architecture for electrode materials of supercapacitors, with high density Ni/NiO core-shell nanospheres dispersing on graphene sheets homogeneously. In this structure, the nickel nanospheres were not completely covered by the shell of nickel oxide due to the parts connecting to graphene sheet was protected by the intense reduction of carbon. Such a deliberately designed structure has several advantages. (1) NiO *in-situ* oxide in Ni nanoparticles possess excellent structural stability, successfully inhibited the copolymerization between nanoparticles. (2) The connection between Ni cores and graphene facilitate rapid export of electrons, and constructs an interconnected 3D conductive network, using metal Ni cores to bridge electrochemical active NiO shell and conductive graphene sheets. (3) The high density Ni/NiO core-shell nanospheres distributed on graphene provide plenty of electrochemical active sites. (4) The porosity among local high density areas (LHDAs) and the small pores among the nickel particles can create different sizes of channels to benefit the OH^−^ ions diffusion and the occurrence of redox reaction. (5) This design is cost-effective. Such a 3D NiO/Ni/RGO core-shell composite was achieved by a simple hydrothermal and heat treatment method. The composites were characterized and their electrochemical performances were measured to estimate their potential ability as the electrode materials for supercapacitors.

## Results

**Figure 1:**
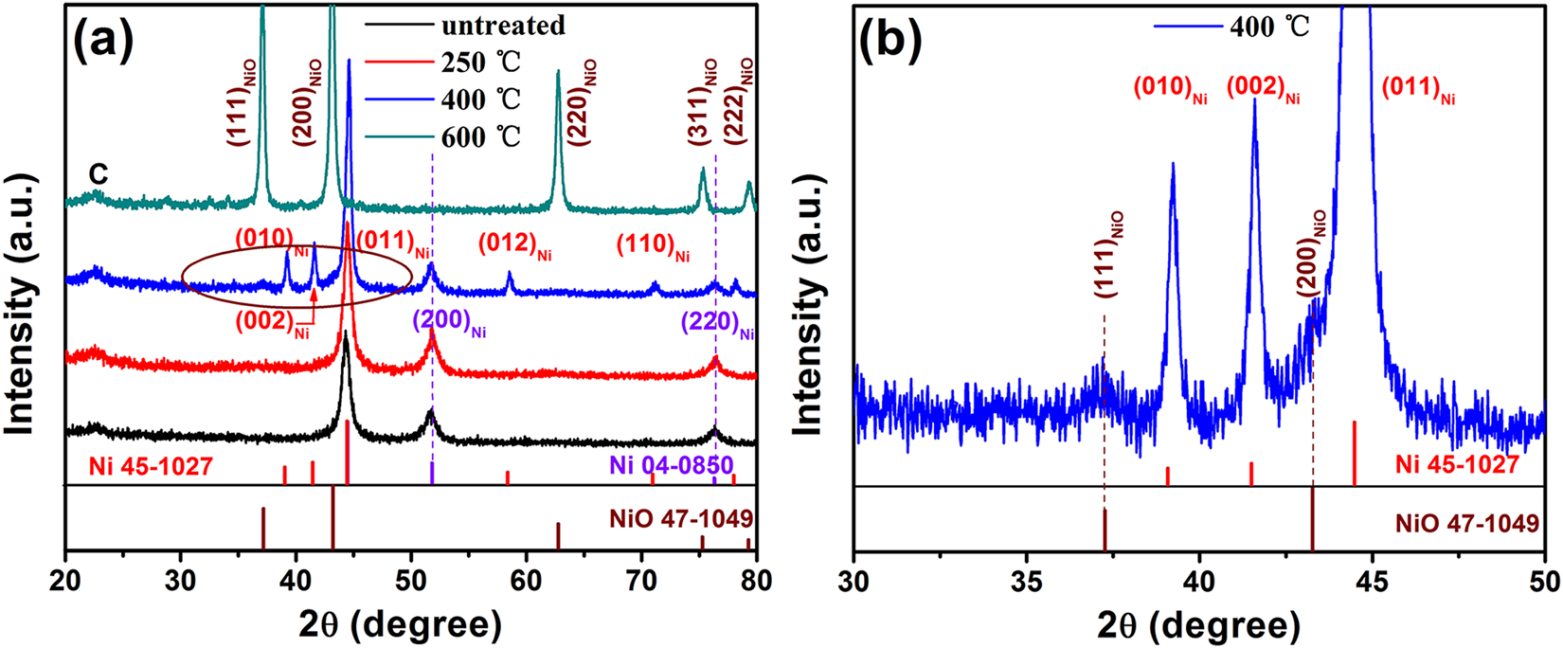
XRD pattern of (**a**) composites with different heat treatment temperature (untreated, 250, 400 and 600 °C) and (**b**) partial enlargement of brown oval in Fig. 1a.

Based on the thermo gravimetric (TG) test in Figure S1, samples based on various temperatures were synthesized, and the corresponding X-ray diffraction (XRD) measurement results were shown in Fig. 1a. The peak centered at around 2θ = 22° always exists for all composites at different temperature, which is the typical peak of graphene C. For the composite without heat treatment and that treated at 250 °C, the characteristic peaks centered at 44.5° is corresponding to the (011) plane of Ni (04-0850), and the peaks centered at 51.8°, and 76.4°are corresponding to the (200) and (220) planes of Ni (45-1027), that is two similar fcc Ni coexists in the structure, and no oxidation of Ni to NiO occurs at 250 °C. When heat treated at 600 °C, all the peaks of Ni disappear and the newly emerging peaks centered at 37.2°, 43.5°, and 62.3° etc. correspond to the (111), (200) and (220) crystal planes of NiO, respectively^16^, which signifies that all Ni had been oxide to NiO at such high temperature. While treated at 400 °C (NiONiG-400-2), as revealed by the enlarged picture shown in Fig. 1b, only a limited part of Ni was oxide to NiO, testified by the slight emerging of (111) and (200) peaks of NiO. So, treated at 400 °C for (NiONiG-400-2) gives the composite we expected. Meanwhile, the peaks of nickel (Ni-2 (45-1027)) became more evident.

**Figure 2:**
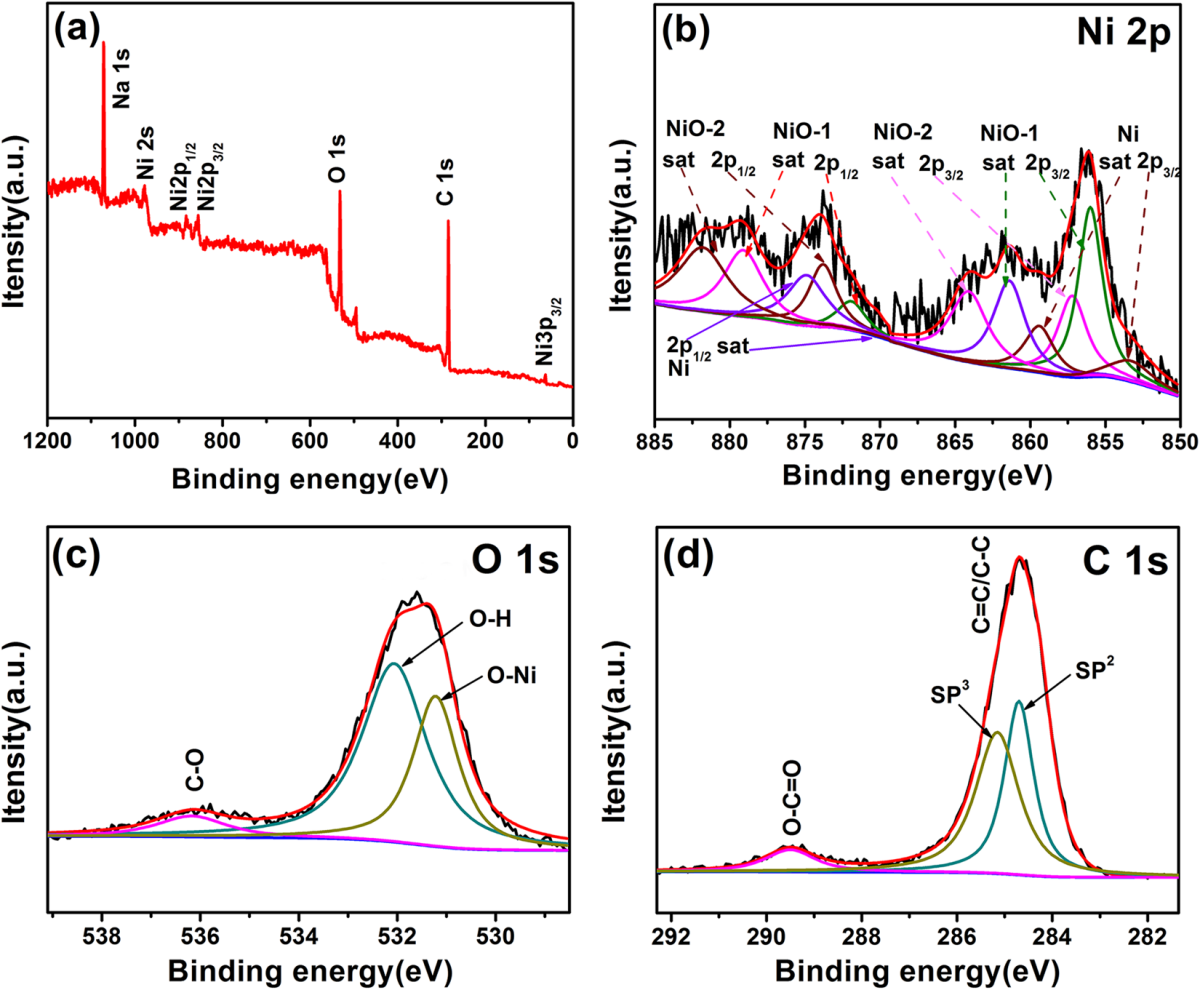
XPS spectra of NiONiG-400-2 composite: (**a**) survey scan, (**b**) Ni 2p, (**c**) O 1 s, and (**d**) C 1 s.

X-ray photoelectron spectroscopy (XPS) spectra of the as-synthesized NiONiG-400-2 composite were shown in Fig. 2. Based on the survey XPS spectrum (Fig. 2a), in addition to a bit Na from the reductant sodium borohydride, the spectra of three elements of Ni, O and C are observed, which well matched with XRD results in Fig. 1. Therein, the Ni 2 s peak appeared at around 1008 eV confirms the existence of metal Ni. Figure 2b shows the Ni 2p spectrum emission, the binding energies located at 871.9 and 873.8 eV are corresponding to the 2p1/2 spin–orbit levels of NiO-1 and NiO-2, while those at 856 eV and 857.2 eV are attributed to their the 2p3/2 spin–orbit levels, respectively. Meanwhile, the 2p1/2 and 2p3/2 spin–orbit levels of Ni centered at 870.1 and 853.5 eV^6^. The presence of two NiO (NiO-1 and NiO-2) is due to the length of Ni-O bond would changed in the oxidation process. In fact, this is the same kind of NiO. Figure 2c shows the characteristic spectrum emission of O 1 s. The binding energy at 531.8 eV can be simulated by two peaks, one at 531.3 eV is attributed to Ni-O and the other at 532.3 eV is attributed to O-H bond^28^. The small binding energy peak located at 535.7 eV is corresponding to C-O. The existence of Ni-O and C-O bonds may mean that both Ni and graphene oxide respectively at the some environment, which may benefit the closely integrated of them. Figure 2d shows the C 1 s spectrum emission of different functional groups, the characteristic peaks of C=C/C-C, O-C=O located at 284.7 and 289.5 eV, respectively^6,29^. C=C and O-C=O can be regarded as the characteristic functional group of reduced graphene oxide, which proved that the graphene structure was well maintained after 400 °C heat treatment. In addition, no other C-O and C=O related to graphene oxide can be fitted, verifying that graphene oxide is well reduced and consistent with the results of Raman tests in Figure S2. In summary, all these results confirm the configurations of graphene, NiO and metal Ni in NiONiG-400-2 samples, being coincident with the XRD results.

**Figure 3:**
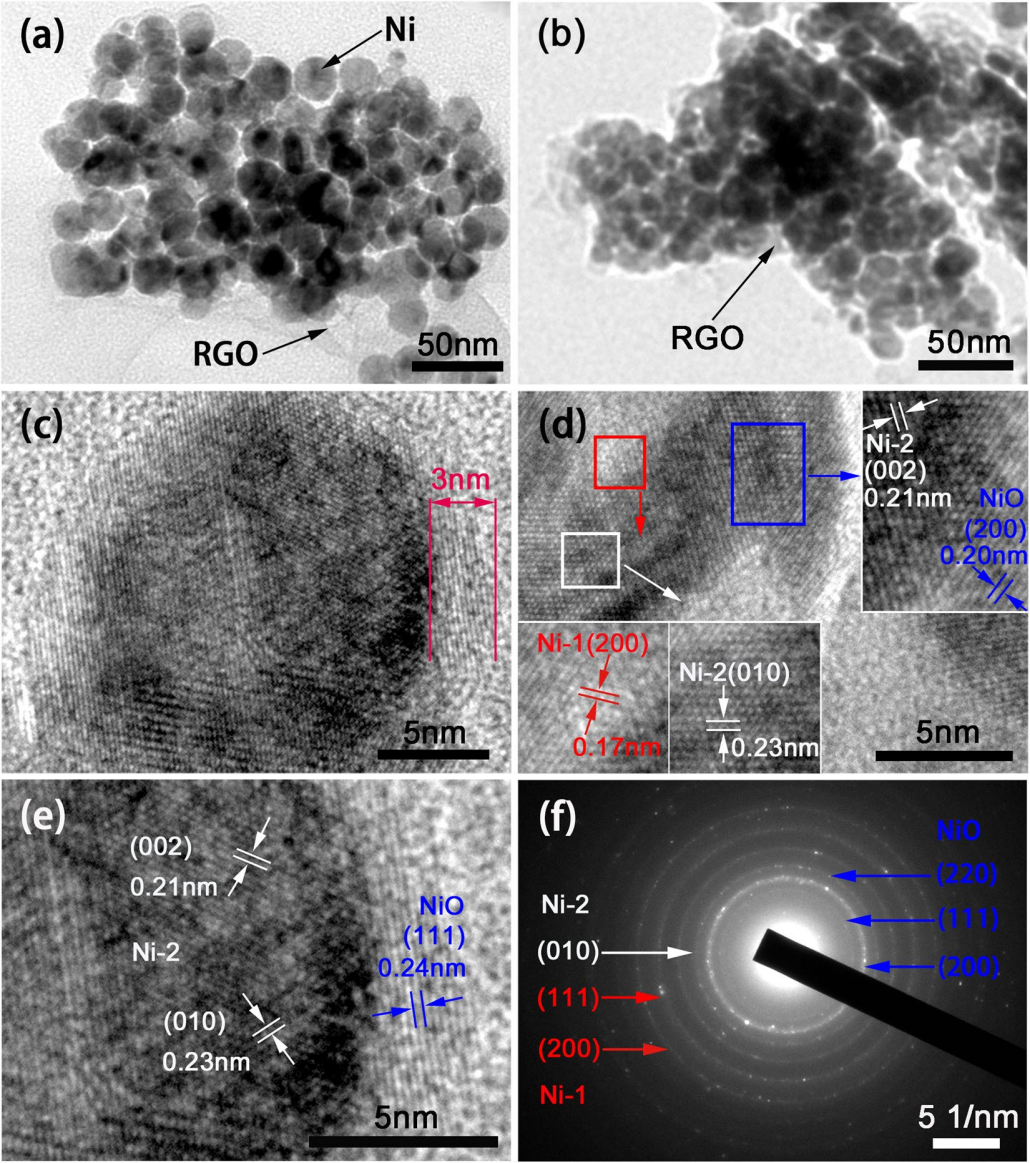
(**a**,**b**) TEM images of RGO/Ni (S-untreated) and NiONiG-400-2, (**c**,**d**) the HRTEM images of NiONiG-400-2 composite, (**e**) partial enlarged drawing of Figure c, (**f**) is corresponding to SAED pattern of NiONiG-400-2 composite.

The microstructures of RGO/Ni (S-untreated) and NiONiG-400-2 composite were investigated with transmission electron microscopy (TEM). Figure 3a shows the TEM image of RGO/Ni, high density nickel nanoparticles of about 20 nm in diameter were uniformly distributed on a graphene sheet. This homogeneous distribution and fully cover of Ni/NiO core-shell on graphene sheet may be attributed to the successful carboxylation of the graphene surface to obtain a large number of functional groups. Meanwhile, diffusion channels with different sizes formed among the stacking nickel nanoparticles, which will facilitate the diffusion of OH^−^ ions and promote the redox reactions^30^. Figure 3b depicts the TEM image of NiONiG-400-2, after oxide at 400 °C, the dispersion and high density characteristics of Ni/NiO nanoparticles on graphene were maintained. A HRTEM image of NiONiG-400-2 composite is illustrated in Fig. 3c to show the core-shell structure. The central dark sphere with a diameter of about 20 nm is the core, coated by a ring of about 3 nm in thickness. Figure 3e gives local enlarged views of the core-shell regions, which clearly shows that the dark core is nickel and the light color shell is nickel oxide. Another local bonding region is shown in Fig. 3d, the atom level interface connection between Ni core and NiO shell is revealed, the interplanar spacing of 0.21 and 0.20 nm are corresponding to the (002) plane of Ni and the (200) plane of NiO, respectively, confirming the continuous transition or coherent bonding of the two phases (Ni and NiO). In the other two enlarged views labeled by red and white rectangles, the observation of two nickel crystals confirmed the coexistence of two crystalline configurations of nickel (Ni-1 (04-0850) and Ni-2 (45-1027)) in the composite. Figure 3f shows selected-area electron diffraction (SAED) image taken on a few dozen core-shell nanoparticles. The mixture of Ni and NiO rings proves that all nanoparticles are coexisting with Ni and NiO^31^, which are good in accordance with the XRD result. And the continuous rings without large light spot indicate the homogeneous size of these nanoparticles.

**Figure 4:**
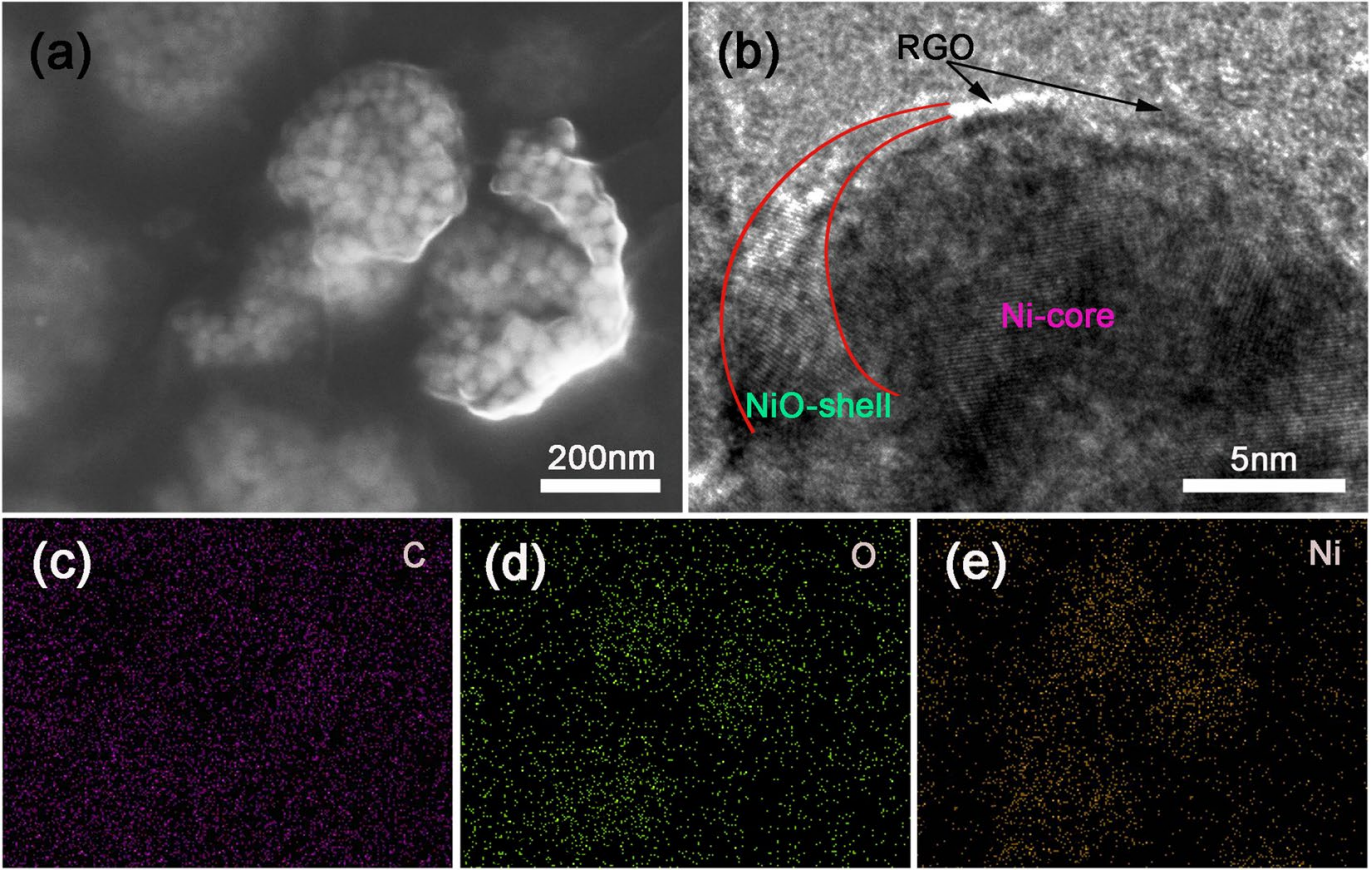
(**a**,**b**) The HRTEM and FESEM images of NiONiG-400-2, (**c**–**e**) surface swept energy spectrum of Figure d.

A FESEM image of NiONiG-400-2 is shown in Fig. 4a; presenting the relatively lower magnification scene of the composite shown in Fig. 3a and b. The corresponding map-scanning surface energy spectrum results are given in Fig. 4c–e, to show the even distribution of elements Ni, O and C. The high density NiO/Ni nanoparticles without aggregation were wrapped up by graphene and formed many local high density areas (LHDAs) that connected to each other. The porosity among LHDAs and the small pores among the nickel particles can provide different sizes of channels to benefit the OH^−^ ions diffusion and improve the electrochemical performance^30^. After tested by N_2_ adsorption-desorption isotherms (Figure S3), the pore diameter of the as synthesized NiONiG-400-2 is mainly distributed at around 2–20 nm, and the more detailed description of LHDAs is shown in Figure S4. The HRTEM image (Fig. 4b) cut from the edge of NiONiG-400-2 sample, shows the coexistence of three components Ni, NiO and graphene. It is clearly seen that the existence of graphene causes the semi-coated NiO/Ni core-shell structure.

## Discussion

**Figure 5:**
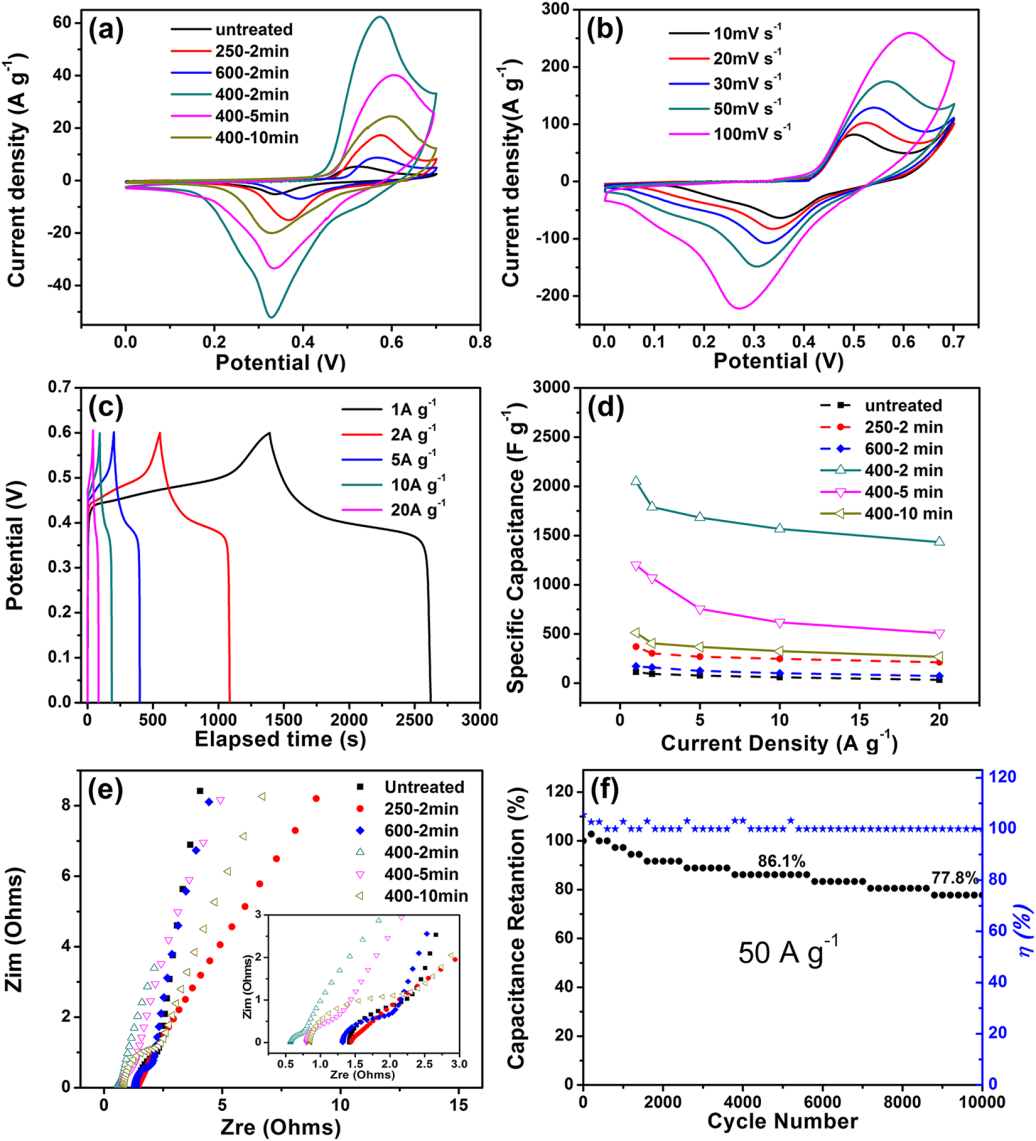
(**a**) CV curves of composites with a scan rates of 10 mV s^−1^ over the voltage range 0 to 0.7 V; (**b**) CV curves of NiONiG-400-2 with different scan rates; (**c**) charge/discharge curves of NiONiG-400-2 at various current densities; (**d**) the specific capacitance of composites with different current densities; (**e**) EIS of composites; (**f**) coulomb efficiency and cycling performances of NiONiG-400-2 at current density of 50 A g^−1^.

The electrochemical performances of the obtained composites used as work electrodes in a three electrode test system are shown in Fig. 5. The CV curves of the composite treated at different temperature were measured at a scan rate of 10 mV s^−1^ over the voltage range from 0.0 to 0.7 V and shown in Fig. 5a The NiONiG-400-2 sample shows the best performance among the composites, exhibited by its largest encircled area or the highest height of the redox peaks. This proves that the formation of the Ni/NiO core-shell structure on graphene sheet is vital to enhance the electrochemical performance. Based on the advantages of the material and structure, and using our unique surface modification method and precise control of the thickness of the oxide layer, a semi-coated core-shell structure with high density nanoparticles uniformly distributed on graphene was prepared. This unique structure may have three advantages: ***a***, high density Ni/NiO nanoparticles provide a large number of the active sites and suppress the agglomerations of both graphene sheets and Ni/NiO nanoparticles themselves. ***b***, semi-coated structure well balance the conductivity and structural stability and the tight connections between graphene and nickel core as well as between nickel core and nickel oxide shell promote the export and transport of electrons among these substances;^16,20^ ***c***, the porosity among LHDAs and the small pores among the nickel particles can provide different sizes of channels to benefit the OH- ions diffusion. Meanwhile, in view of the limited infiltration of the electrolyte, the thickness of the oxide layer has an important impact onto the electrochemical properties. The NiONiG-400-2 composite should have a good balance between the number of active sites and the utilization of active materials by appropriately controlling the temperature and time of oxidation. Prolonging the oxidation time to 5 min or 10 min will thicken the NiO shell and make the nanoparticles adhering to each others, which would reduce the active sites and block the ion diffusion channel. So, a decrease of encircled area with increasing heat treating time is observed. The redox peaks (existing around 0.55 and 0.32 eV) indicate the revisable redox reaction occurred as followed^32^:

1 $$NiO+O{H}^{-}\leftrightarrow NiOOH+{e}^{-}$$

The rate capability of NiONiG-400-2 electrode material was evaluated by CV and galvanostatic charge–discharge tests. Figure 5b illustrates the CV curves of the NiONiG-400-2 sample with different scan rates (10, 20, 30, 50 and 100 mV s^−1^). The retention of the shape in different scan rates indicates good rate capability of the electrode material, while the shift of redox peaks is attributed to the polarization^33,34^. A further examination of rate capability was carried out at a serious of current densities (1, 2, 5, 10 and 20 A g^−1^) shown in the Fig. 5c. The discharge time decreases rapidly with increasing the current density, as there is no enough time for the penetrating of electrolyte ions into the deep pores of the electrode material at high current density. Different from the linear charge/discharge behavior of electric double layer capacitors, NiONiG-400-2 electrode shows typical pseudocapacitive characteristic. In general, the specific capacitance of the electrode material can be calculated from charge–discharge curves according to the equation as followed:

2 $${C}_{s}=(I\cdot {\rm{\Delta }}t)/(m\cdot {\rm{\Delta }}V)$$

Where *I* the discharge is current, Δ*t* is the discharge time, *m* is the mass of the electrode, and Δ*V* is the voltage window, respectively. Figure 5d demonstrates the comparison of rate performance for composites with different treat temperature and time at a serious of current densities. On the basis of the discharge curves, the specific capacitances of NiONiG-400-2 are 2048.3, 1790, 1683.3, 1566.7, and 1433.3 F g^−1^, respectively, corresponding to current densities of 1, 2, 5, 10 And 20 A g^−1^. Chundong W *et al.* ^9^ by hydrothermal means prepared hierarchical NiO-3D graphene composite delivers a high specific capacitance of 1829 F g^−1^ at a current density of 3 A g^−1^, which fully illustrates that NiO/graphene composites have sufficient potential in the preparation of high performance supercapacitor. Xinyi H *et al.* ^35^ prepared core/shell structure of NiCo_2_O_4_@NiCoAl-LDH onto nickel foam and displays a specific capacitance of 1814.24 F g^−1^ at a current density of 1 A g^−1^. These performances have a significant advantage over other structures, thus demonstrating that the core-shell structure has its own distinct superiority in the preparation of high performance capacitors. As compared with previous studied NiO based supercapacitors shown in Table 1. We based on the advantages of the above materials and structures, and using our unique surface modification method and precise control of the thickness of the oxide layer, a semi-coated core-shell structure with high density nanoparticles uniformly distributed on graphene was prepared. The present NiO/Ni/RGO composite shows the highest specific capacitance values among NiO electrodes at corresponding current density, the present highest value of 2048.3 F g^−1^ reaches to about 80% of the theoretical value of capacitance (2584 F g^−1^) of NiO.

**Table 1:** Electrochemical performance of NiO-based Supercapacitors.

Specimen structure	Tested current density [A g^−1^/mV s^−1^]	Specific capacitance [F g^−1^]	Cycling number	Capacity retention [%]	Year Published	Ref.
3D UGF/CNTs/NiO	1 A g^**−**1^	750.8 F g^**−**1^	3000	100%	2014	6
Hierarchical NiO-3D Graphene	3 A g^**−**1^	1829 F g^**−**1^	5000	85%	2014	9
3D NF-G-NiO	5 A g^**−**1^	950 F g^**−**1^	—	—	2016	15
CO_3_O_4_/NiO core-shell nanowires arrays	2 A g^**−**1^	853 F g^**−**1^	—	—	2011	17
3D porous RGO @ NiO	1 A g^**−**1^	1328 F g^**−**1^	2000	87%	2014	14
NiO/Graphene	5 mV s^**−**1^	816 F g^**−**1^	2000	100%	2011	27
CNTs @ NiO core-shell	1 A g^**−**1^	996 F g^**−**1^	10000	93%	2015	16
Pt & NiO/Ni core-shell	1 A g^**−**1^	900 F g^**−**1^	—	—	2011	20
High density NiO/Ni/RGO core-shell	1 A g^**−**1^	2048.3 F g^**−**1^	5000	86.1%	Present work	
			10000	77.8%		

**Figure 6:**
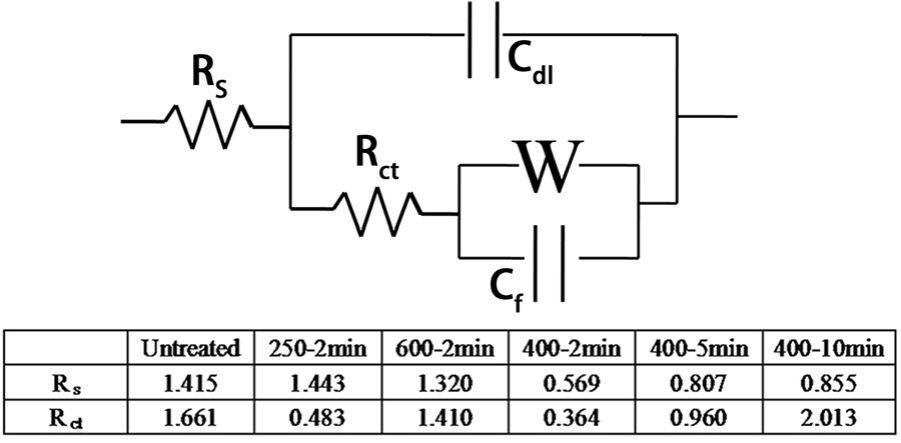
Equivalent circuit and impedance data.

The electrochemical impedance spectra (EIS) measurements for the composites with different treat temperatures and times were carried out from100 KHz to 0.01 Hz with alternate current amplitude of 5 mV in 1 M KOH electrolyte and the results are shown in Figs 5e and 6 is the equivalent circuit and impedance data. The intercept on the real axis indicates the equivalent series resistance (R_s_) and the semicircle in the high frequency region reflects the interface charge transfer resistance (R_ct_) between the electrode and electrolyte, and W, C_dl_, C_f_ are the Warburg impedance, double layer capacitance, and limit capacitance, respectively. Based on the enlarged view of high frequency region in Fig. 5e and the table in Fig. 6, NiONiG-400-2 electrode exhibits the smallest R_s_ and R _ct_, which indicates that the NiO/Ni/RGO core-shell structure have excellent electron transfer ability and can provides lots of diffusion channels for ions, these are necessary advantages to ensure the ultrahigh specific capacitance of the NiO/Ni/RGO composite.

Cycling stability is an important index value to evaluate the electrochemical performance of supercapacitors. Yi, H *et al.* ^16^ developed a novel ASC (asymmetric supercapacitors) based on carbon nanotube @ nickel oxide nanosheets (CNT @ NiO) core-shell composites for supercapacitor with 93% capacitance retention after 10000 cycles, which shows the excellent stability of the nickel oxide material as the electrode material. The cycling performance of NiONiG-400-2 was tested in the three electrode system at current density of 50 A g^−1^, shown in Fig. 5f. The specific capacitance slowly decreases with increasing the cycles, which may be ascribed to the exfoliation of electrode material from Ni foam during the cycling process. Even though, the electrode material maintains 86.1% capacitance retention after 5000 cycles and 77.8% capacitance retention after 10000 cycles in the meantime, exhibiting an excellent cycle stability. The inhibitory effect for graphene agglomeration by high density nanoparticles and good maintain of core-shell frame structure are two main reasons for good cycle stability.

## Conclusion

A NiO/Ni/RGO composite applying for electrode materials of supercapacitors was fabricated by a simple hydrothermal plus heat treatment method. The composite has a 3D structure consisting of Ni/NiO core-shell nanoparticles evenly distributed on the graphene sheets. This unique NiO/Ni/RGO 3D core-shell architecture, using metal Ni cores to bridge electrochemical active NiO shell and conductive graphene sheets, forms a continuous 3D conductive network in the structure and provides a continuous channel for ion diffusion through the gaps and pores among the Ni/NiO core-shell nanoparticles. Therefore, the electrochemical tests demonstrate that the prepared composite possess a very high specific capacitance of 2048.3 F g^−1^ at current densities of 1 A g^−1^, about 80% of the theoretical capacitance of NiO (2584 F g^−1^) and the highest specific capacitance value ever obtained for NiO based materials. This composite also maintains good cycle stability (77.8% capacitance retention after 10000 cycles). From a methodological point of view, the novel method may provide reference for the preparation of other high performance supercapacitor.

## Methods

### Fabrication of RGO/Ni composites

RGO/Ni composites were synthesized by redox reactions with further hydrothermal method. Firstly, a 2 mg mL^−1^ graphene oxide aqueous solution, a sodium hydroxide solution of Ph = 13 and a 40 mg mL^−1^ chloroacetic acid aqueous solution were prepared, respectively. The graphene oxide aqueous solution was ultrasound stirred for two hours, then slowly dropwise the sodium hydroxide solution into graphene oxide aqueous solution with volume proportion of 5: 6, stir for 30 minutes for homogeneous mixture. The pH of the mixture solution was adjusted to be 11 with the above mentioned chloroacetic acid aqueous solution, ultra sounding for one hour and stirring overnight. Then, the as-prepared solution was dialyzed until pH was 7. Add 120 mg Polyvinylpyrrolidone into 30 mL as-prepared solution to prevent graphene oxide agglomeration with the metal salt solution. Afterword, a certain amount of nickel sulfate (NiSO_4_: GO = 1: 8) was dissolved in 10 mL deionized water, and added it slowly dropwise into the above-mentioned solution, stirring for 1 hour. Then, add sodium borohydride solution (600 mg dissolved in 10 mL deionized water) into above-mentioned as-prepared solution slowly. Finally, as-prepared solution was added into the autoclave for hydrothermal reaction at 180 °C for 12 hours followed by natural cooling to room temperature. The synthesized composites were washed with deionized water and alcohol for several times, and dried in vacuum atmosphere at 60 °C, and was labeled as S-untreated (precursor).

### Synthesis of NiO/Ni/RGO composites electrode materials

**Figure 7:**
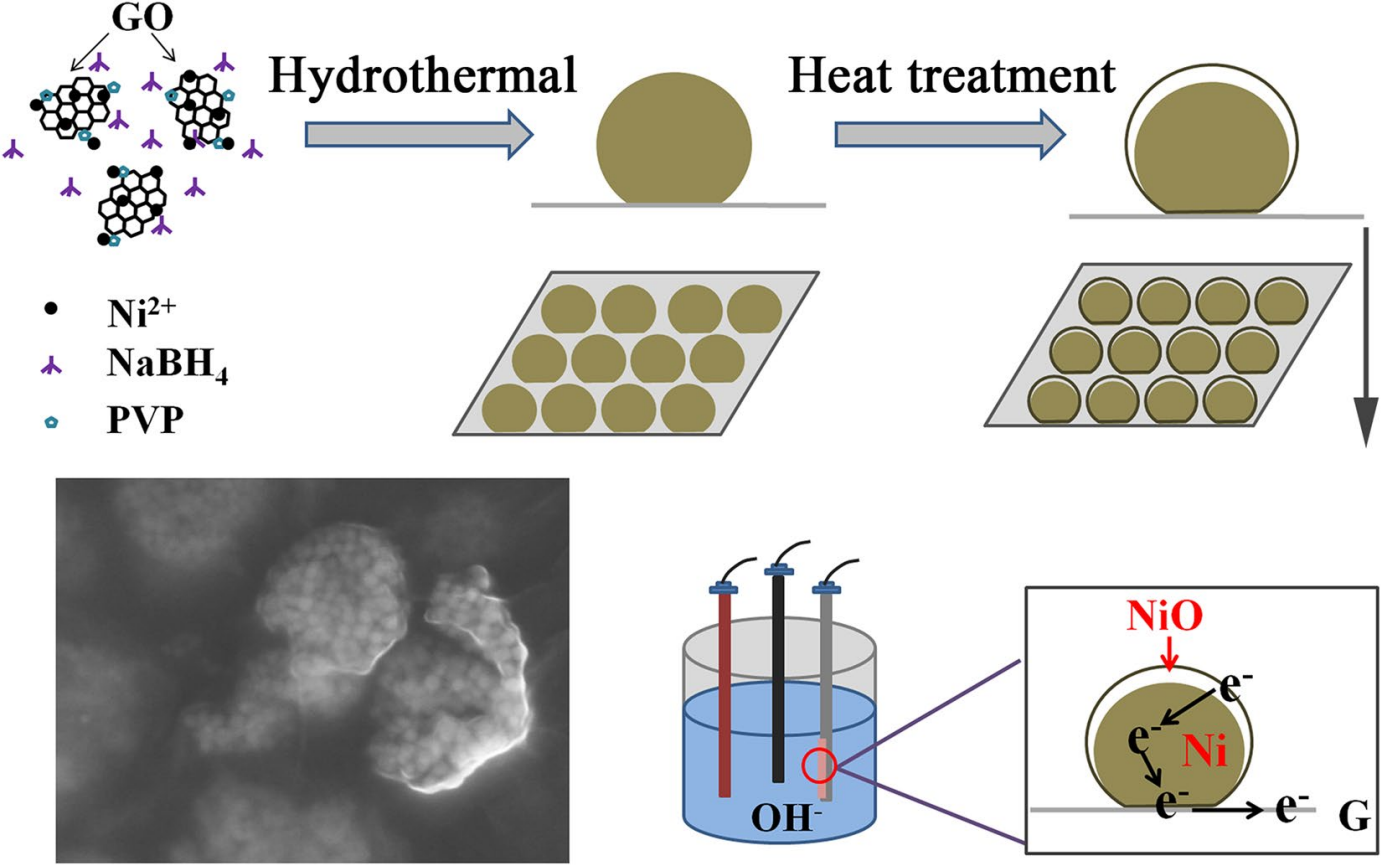
The schematic diagram of preparing core-shell structure and its conductive mechanism.

In order to form a NiO layer on the Ni surface, the precursor was subjected to heat treatment at different temperatures for different times. The above mentioned S-untreated precursor was treated at 400 °C for 2 min at air atmosphere with a 5 °C temperature increase rate. After naturally cooled to room temperature, the as obtained sample was labeled as NiONiG-400-2. For comparison, analogous methods were used to treat the precursors at temperature of 250 and 600 °C for 2 min, and 400 °C for 5 and 10 min, respectively. The corresponding samples were labeled as NiG-250-2, NiOG-600-2, NiONiG-400-5 and NiONiG-400-10. The schematic diagram of preparation process is shown in diagram Fig. 7.

### Characterizations

The crystal structures of the composites were revealed by X-ray diffraction (XRD) using a Rigaku D/max 2500PC diffractometer with Cu K_α_ radiation (λ = 1.54156 Å), recorded with the 2θ ranging from 20 to 80° at a scanrates of 4°min^−1^. The thermo gravimetric analysis (TGA) of NiONiG-400-2 was done in NETZSCH STA 449F3. The weight loss of the samples was monitored from room temperature to 700 °C at a heating rate of 10 °C min-1 in nitrogen atmosphere. Raman spectra were obtained on a 514 nm laser beam. The specific surface area was calculated by the Brunauer–Emmett–Teller (BET) method, and pore size distribution was obtained from desorption plots by a Barrett–Joyner–Halenda (BJH) analysis. The field emission scanning electron microscope (FESEM, JSM-6700F) and transmission electron microscope (TEM, JEM-2100F) were used to observe the microscopic morphology of the as-prepared samples. X-ray photoelectron spectroscopy (XPS) was used to determine the elements and chemical states in the samples, which was realized on an ESCALAB Mk II (Vacuum Generators) spectrometer with unmono-chromatized Al K_α_ X-rays (240 W).

### Electrochemical measurements

For preparing electrode, the as-prepared composites, acetylene black and polyvinylidene fluoride at a ratio of 80: 15: 5 were manually grinded adequately for 3 hours with appropriate amount of methylpyrrolidone as mixed solvent, then the slurry were smeared on Ni foam uniformly and dried at 80 °C for 12 hours. The electrochemical performances of the NiONiG-400-2 composites as work electrodes were characterized by a PMC-1000 electrochemical workstation in a three-electrode system using calomel electrode as reference electrode and Pt sheet electrode as counter electrode with 1 M KOH aqueous solution as the electrolyte. Cyclic voltammograms (CV) with the potential window from 0 to 0.7 V at different scanrates varying from 10 to 100 mV s^−1^, galvanostatic charge–discharge with the constant current density ranging from 1 to 50 A g^−1^ and electrochemical impedance spectroscopy (EIS) with the constant current density of 50 A g^−1^ for 10000 cycles were measured in sequence.

## Electronic supplementary material

Supplementary Information
