# Supplementary material for: High Density Arrayed Ni/NiO Core-shell Nanospheres Evenly Distributed on Graphene for Ultrahigh Performance Supercapacitor
Source: Sci Rep. 2017 Dec 18;7:17709. doi: 10.1038/s41598-017-17899-6 (PMC5735128; doi:10.1038/s41598-017-17899-6)
Supplement: Supplementary file 1 — Supplementary Information [file 41598_2017_17899_MOESM1_ESM.doc]

**Supporting Information**

**High Density Arrayed Ni/NiO Core-shell Nanaospheres Evenly Distributed on Graphene for Ultrahigh Performance Supercapacitor**

Fanggang Liu ^1†^, Xiaobing Wang^1†^, Jin Hao^†^ , Shuang Han*^†^, Jianshe Lian*^†^, Qing Jiang^†^

† Key Laboratory of Automobile Materials, Ministry of Education, and Department of Materials Science and Engineering, Jilin University, Changchun 130022, P.R. China


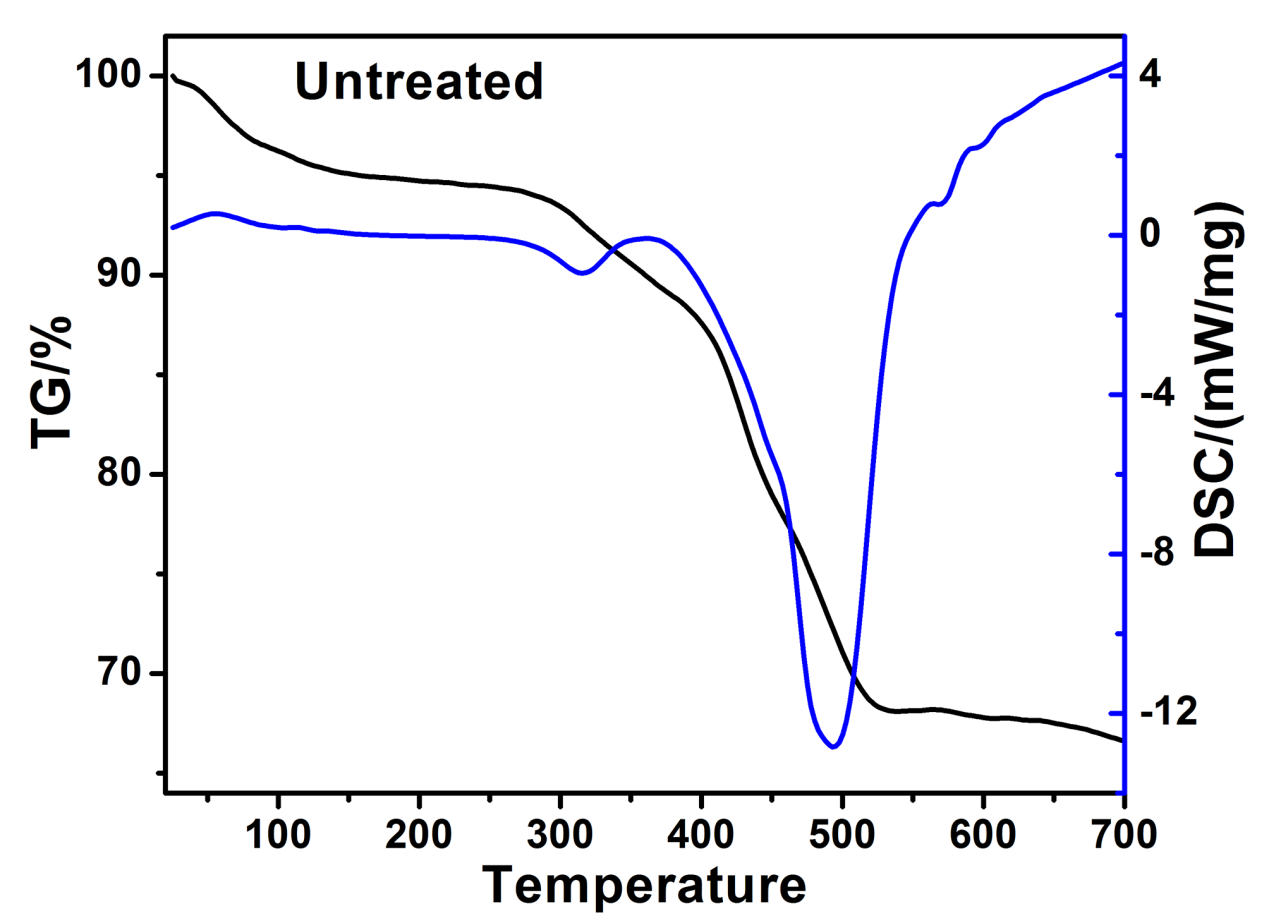


**Figure S1 TG curve of the precursor.**

From TG curve of the precursor shown in Figure S1, before 100 ℃, the precursor began to have some weight loss, which is due to the removal of moisture in the material. The slow mass loss of GO at 100℃ to 300 ℃ is caused by the decomposition of the oxide functional groups. Meanwhile, the weight loss is the comprehensive result of oxidation of Ni and combustion of graphene at the temperature of 300℃ to 700 ℃. From 300℃ to 400℃, the rate of weight loss is slowed down due to the dominance of Ni oxidation, and referred to TG curve of GO in previous reports,^1^ it can be concluded that there is still remaining of graphene after 600 ℃, which is consistent with the characteristic peaks of carbon in our XRD.


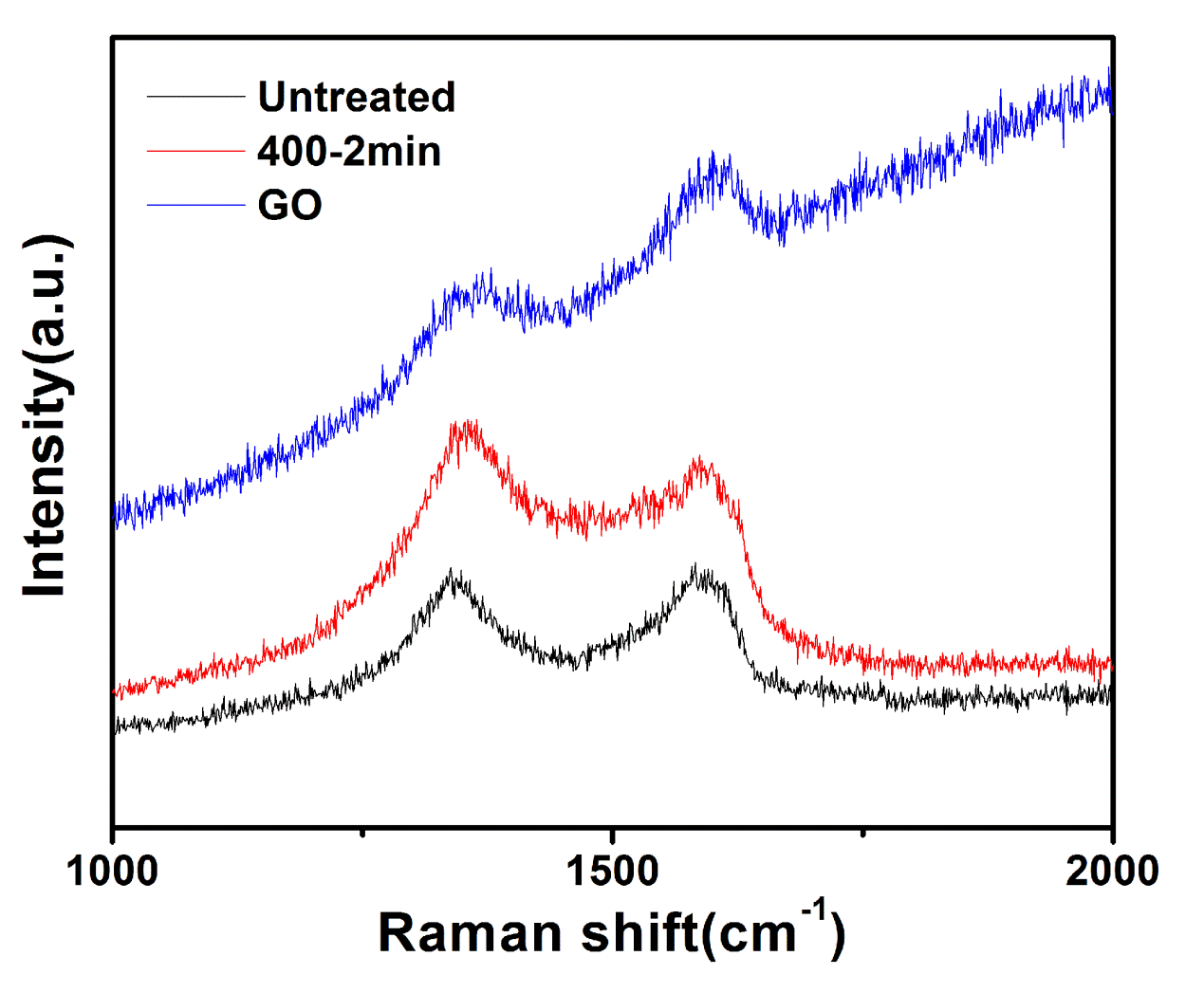
 **Figure S2 Raman spectra of GO, precursor and NiONiG-400-2.**

The Raman spectra of GO, precursor and NiONiG-400-2 are shown in Figure S2, and the spectra display the carbon D and G band peaks at ∼1350 cm^−1^ and 1580 cm^−1^, respectively. Generally, the D band originating from defects such as vacancies, grain boundaries, and amorphous carbon species is an indication of substantial disorder in the graphene sheets. ^2^ The D/G intensity ratio precursor and NiONiG-400-2 are increased to 0.98 and 1.03 compared to the 0.81 of GO, inferring that more defects formed when some oxygen atoms are removed. ^3^ Especially, the D/G intensity ratio of NiONiG-400-2 has a further increase to precursor indicating the more thorough reduction of GO after heat treatment.


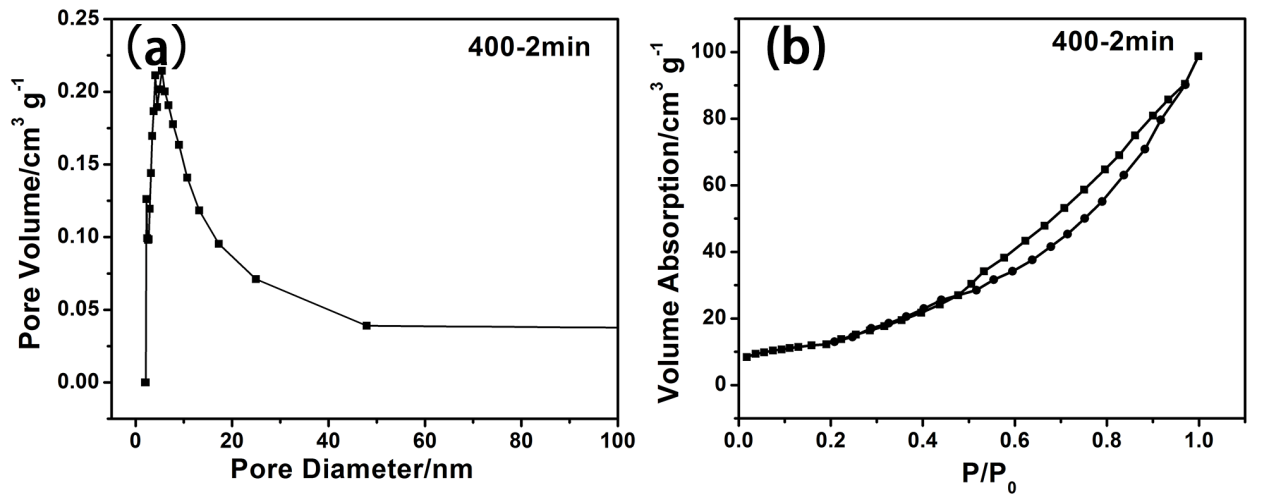
 **Figure S3 (a, b) Pore size distribution patterns and** [**Nitrogen**](javascript:popupOBO('CHEBI:25555','C1JM13016A','http://www.ebi.ac.uk/chebi/searchId.do?chebiId=25555'))**adsorption/desorption isotherms of NiONiG-400-2.**

N_2_ adsorption-desorption isotherms and the pore size distribution plots of NiONiG-400-2 are shown in Figure S3. According to International Union of Pure and Applied Chemistry (IUPAC) classification, isotherms of NiONiG-400-2 display type IV with H3 type hysteresis loop. The pore diameter is mainly distributed at around 2–20 nm and the BET surface area of NiONiG-400-2 is 52.8 m^2^ g^−1^. These results can be ascribed to the high density NiO nanoparticles are dispersed uniformly on the graphene, which can prevent the graphene from aggregating and provide an efficient transport pathway for electrolyte.


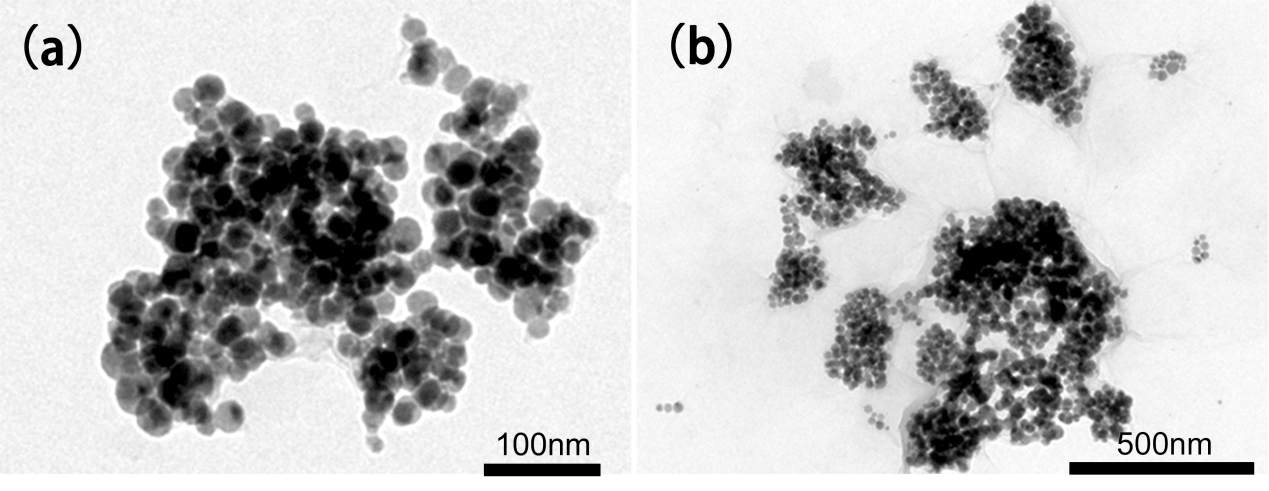


**Figure S4 TEM images of NiONiG-400-2.**

The relative low-magnification TEM images of NiONiG-400-2 are shown in Figure S4. In Figure S4 (a), the high density nickel nanoparticles are evenly distributed on the graphene after the heat treatment. Due to the surface modification of graphene, clusters of nickel nanoparticles (LHDAs) with homogeneous high density are formed on graphene, as shown in Figure S4 (b). Although these LHDAs are separated from each other, they are interconnected by graphene, which ensuring the electrolyte rapidly diffused through the channel and allowing electrons transfer through graphene conveniently between them.

**Table 1.** Electrochemical performance of NiO-based Supercapacitors.

| **Specimen structure** | **Tested current density**  **[A g^−1^ / mV s^−1^]** | **Specific capacitance**  **[F g^−1^ ]** | **Cycling**  **number** | **Capacity retention**  **[%]** | **Year**  **Published** | **Ref.** |
| --- | --- | --- | --- | --- | --- | --- |
| **3D UGF/CNTs/NiO** | 1 A g^−1^ | 750.8 F g^−1^ | 3000 | 100% | 2014 | 6 |
| **Hierarchical NiO-3D Graphene** | 3 A g^−1^ | 1829 F g^−1^ | 5000 | 85% | 2014 | 9 |
| **3D NF-G-NiO** | 5 A g^−1^ | 950 F g^−1^ | ─ | ─ | 2016 | 15 |
| **CO_3_O_4_/NiO core-shell nanowires arrays** | 2 A g^−1^ | 853 F g^−1^ | ─ | ─ | 2011 | 17 |
| **3D porous RGO @ NiO** | 1 A g^−1^ | 1328 F g^−1^ | 2000 | 87% | 2014 | 14 |
| **NiO/Graphene** | 5 mV s^−1^ | 816 F g^−1^ | 2000 | 100% | 2011 | 27 |
| **CNTs @ NiO core-shell** | 1 A g^−1^ | 996 F g^−1^ | 10000 | 93% | 2015 | 16 |
| **Pt & NiO/Ni core-shell** | 1 A g^−1^ | 900 F g^−1^ | ─ | ─ | 2011 | 20 |
| **High density**  **NiO/Ni/RGO core-shell** | 1 A g^−1^ | 2048.3 F g^−1^ | 5000  10000 | 86.1%  77.8% | Present  work |  |

**References**

1 Fan, X. *et al.* Deoxygenation of Exfoliated Graphite Oxide under Alkaline Conditions: A Green Route to Graphene Preparation. *Advanced Materials* **20**, 4490-4493, doi:10.1002/adma.200801306 (2008).

2 Zhao, B. *et al.* Supercapacitor performances of thermally reduced graphene oxide. *J Power Sources* **198**, 423-427, doi:10.1016/j.jpowsour.2011.09.074 (2012).

3 Chen, Y., Zhang, X., Zhang, D., Yu, P. & Ma, Y. High performance supercapacitors based on reduced graphene oxide in aqueous and ionic liquid electrolytes. *Carbon* **49**, 573-580, doi:10.1016/j.carbon.2010.09.060 (2011).
